# IHIT-BED: an interpretable transformer approach using unbiased hematology analyzer impedance data for early identification of bacteremia in emergency department

**DOI:** 10.1093/bioadv/vbaf322

**Published:** 2025-12-23

**Authors:** Tung-Lin Tsai, Chien-Chong Hong, Hsing-Wen Cheng, Chin-An Yang

**Affiliations:** Department of Power Mechanical Engineering, National Tsing Hua University, Hsinchu, 300, Taiwan; Department of Laboratory Medicine, Chang Gung Memorial Hospital, Linkou, 333, Taiwan; Department of Power Mechanical Engineering, National Tsing Hua University, Hsinchu, 300, Taiwan; Division of Laboratory Medicine, China Medical University Hsinchu Hospital, Zhubei, 302, Taiwan; Department of Laboratory Medicine, Chang Gung Memorial Hospital, Linkou, 333, Taiwan; Division of Laboratory Medicine, China Medical University Hsinchu Hospital, Zhubei, 302, Taiwan; School of Medicine, College of Medicine, China Medical University, Taichung, 404, Taiwan; Department of Biomedical Engineering and Environmental Sciences, National Tsing Hua University, Hsinchu, 300, Taiwan

## Abstract

**Motivation:**

Early detection of severe bloodstream infections is essential for early treatment initiation. However, the suspicion of bacteremia relies on the combined interpretation of routine laboratory tests, such as complete blood count (CBC), differential count (DC), and elevated C-reactive protein (CRP). Furthermore, a definite diagnosis of bacteremia requires a positive blood culture, which takes several days.

**Results:**

We developed the Interpretable Hematology analyzer Impedance data-based Tabular network for early identification of Bacteremia in Emergency Department (IHIT-BED), a blood stream infection prediction system built by machine learning methods using the integrated data of hematology analyzer impedance histogram signals of CBC, blood culture reports, and CRP levels, which were simultaneously tested in the first blood draw of patients visiting the ED. To our knowledge, IHIT-BED is the first predictor based on hematology impedance histogram signals, which performs well not only in predicting a positive blood culture and severe inflammation, but also is sensitive to detect changes in blood cell morphologies correlated with active inflammatory responses to bacterial infections. IHIT-BED provides clinical decision support for prompt initiation of antibiotics treatment.

**Availability and implementation:**

The method can be found in https://github.com/appleRtsan/IHIT-BED.

## 1 Introduction

Bacteremia refers to the state in which bacteria are present in the bloodstream and is a life-threatening disease. The early detection of bacteremia is essential for the early initiation of treatment. Without early antibiotic care, it can progress to sepsis, which remains a major cause of morbidity and mortality in the world (Raghavan and Marik 2006, Holmes *et al.* 2021). Currently, the majority of bacteremia and sepsis detection still rely on blood culture (BC), which takes several days and is not suitable for the rapid diagnosis of patients at the emergency department (ED) (Vincent *et al.* 2013). Early diagnosis of sepsis may also be challenging due to the risk of blood culture contamination and the complex sepsis-3 criteria (Ntusi *et al.* 2010, Singer *et al.* 2016). The suspicion of bacteremia relies on the combined interpretation of routine laboratory tests such as complete blood count (CBC), differential count (DC), and elevated C-reactive protein (CRP) levels. The parameters of CBC and DC have been suggested to assist in the diagnosis of sepsis (Behrman *et al.* 1981, Huang *et al.* 2020), while CRP levels have been reported to correlate with mortality and organ failure among sepsis patients(Lobo *et al.* 2003), particularly in cases where CRP level exceeds 10 mg/dL (Povoa *et al.* 1998). However, a CRP test takes 20–40 min, and could have false-negative issues, such as the level detected in patients with liver function impairment (Deutsch *et al.* 2018).

In the ED, CBC is the most ordered blood test, which is performed by hematology analyzers via the impedance principle. Hematology analyzers also provide cellular DC parameters via flow cytometry (Hedley *et al.* 2011, Huang *et al.* 2020). Changes in DC and parameters related to blood cell morphologies, such as the presence of leukocyte left shift (Behrman *et al.* 1981), monocyte distribution width (MDW) (Alsuwaidi *et al.* 2022, Crouser *et al.* 2017, 2019), neutrophil degranulation (Camicia *et al.* 2014), and alteration in platelets (Middleton *et al.* 2019), have been reported to be associated with severe bloodstream bacterial infections. However, further examination of DC and blood cell morphologies requires additional test ordering, slide making, and manual microscopic examinations, which are time-consuming. The application of MDW in predicting sepsis is also limited because it is suggested to be used in combination with abnormal white blood cell (WBC) results, and the MDW parameter is not available on every routine hematology analyzer (Crouser *et al.* 2017, 2019).

Impedance histograms of CBC tests are the most commonly acquired data in clinical hematology laboratories. Histograms were generated from the sizes and relative numbers of WBCs, red blood cells (RBC), and platelets (PLT) gated on different volume thresholds. It has been suggested that the impedance histogram contains information about changes in blood cell morphologies, and therefore might be useful in the detection of early pathologies of diseases (Thomas *et al.* 2017a). Thomas *et al.* demonstrated the utility of impedance histograms to provide diagnostically relevant information at early disease stages based on their own experience (Thomas *et al.* 2017a). Dixit *et al.* used the curve shape of impedance data to diagnose childhood anemia, but the utility of the histograms in this study was limited by the interference of cell fragments and bubbles in the flow cells, which created background noise (Dixit *et al.* 2022). Currently, although impedance histograms are widely available on every automated hematology analyzer at routine diagnostic laboratories, the application of WBC, RBC, platelet channels of the histograms for the prediction of early clinical pathologies are still neglected due to the above limitations. Impedance histograms are primarily used for the analysis of cell counts.

Existing prediction models for bacteremia/sepsis utilized different laboratory tests or combining the clinical parameters from the electronic health record (EHR) as inputs for training of the machine learning models, which varied in the accuracy of prediction (Behrman *et al.* 1981, Agnello *et al.* 2024, Amrollahi *et al.* 2025). Single parameter, such as neutrophil-to-lymphocyte count ratio (NLCR), attained an AUC of 0.69 in predicting bacteremia (Abiramalatha *et al.* 2016), while the combination of vital signs and WBC count using a support vector machine (SVM) model reached an AUC of 0.73 in predicting sepsis (Gultepe *et al.* 2014). Li *et al.* (2022) combined DCs, inflammation markers, and vital signs to predict bloodstream infections via machine learning; however, their true positive rate was only 0.62. A study utilizing more than 290 000 CBC/DC data as input achieved an AUC of 0.80 in predicting blood culture results; however, the CBC/DC data were obtained on the same day of blood culture reports, which limited its application on early detection of bacteremia (Lien *et al.* 2022). Amrollahi *et al.* (2025) developed ML models using laboratory and diagnostic information embedded in the EHR of more than 135 000 ED blood culture orders, the best AUC was 0.81, when combining the pre-test diagnostic code as input. There were studies using CBC/DC and cell population data (e.g. MDW) acquired at the time of ED admission to predict bacteremia/sepsis (Lien *et al.* 2022, Chang *et al.* 2023b). MDW alone was reported to discriminate sepsis from other conditions visiting the ED with an AUC of 0.79 in a small cohort of 1320 subjects (Crouser *et al.* 2017). Chang *et al.* developed machine learning models trained with more than 19 600 data obtained at ED admission using CBC/DC and parameters of cell population data acquired via the VCS technology of the hematology analyzer, and achieved AUCs between 0.80 and 0.84 when algorithms such as random forest, logistic regression, and extreme gradient boosting (XGBoost) were applied (Crouser *et al.* 2017, Lien *et al.* 2022, Chang *et al.* 2023a). However, the DC parameter may require manual microscopic examination, and the results of blood smear interpretation may vary between laboratories. Furthermore, cell population data (e.g. MDW) may not be a routine diagnostic test and is dependent on the ordering of physicians. This could contribute to the missing values in the machine learning model inputs, increasing the risk of data drifting in clinical applications (Zhang *et al.* 2008).

Impedance histograms consisted of 256 signals in the WBC, PLT, and RBC channels without missing values. Moreover, impedance signals contain unbiased information reflecting CBC/DC parameters and cell population/morphology changes in response to infection and inflammation. Several deep learning techniques, such as transformers (Jaderberg *et al.* 2015) and self-attention (Vaswani *et al.* 2017) have shown favorable outcomes in handling data or signals that cause mutual channel interference (Cai *et al.* 2022). Notably, the application of transformers in tabular bioinformatics data has been suggested to yield excellent prediction results and explanations (Ko *et al.* 2024). Li *et al.* integrated attention-based graph neural networks, deep tabular learning, and population subgraph partitioning to improve predictive accuracy for disease progression in multimorbid patients using structured medical tabular data (Li *et al.* 2026). Furthermore, application of deep neural models with transformers and interpretability methods has also been reported to better evaluate the importance of sleep and circadian rhythm biomarkers derived from wearable data for detecting metabolic syndrome (Kim *et al.* 2025).

**Figure 1.**
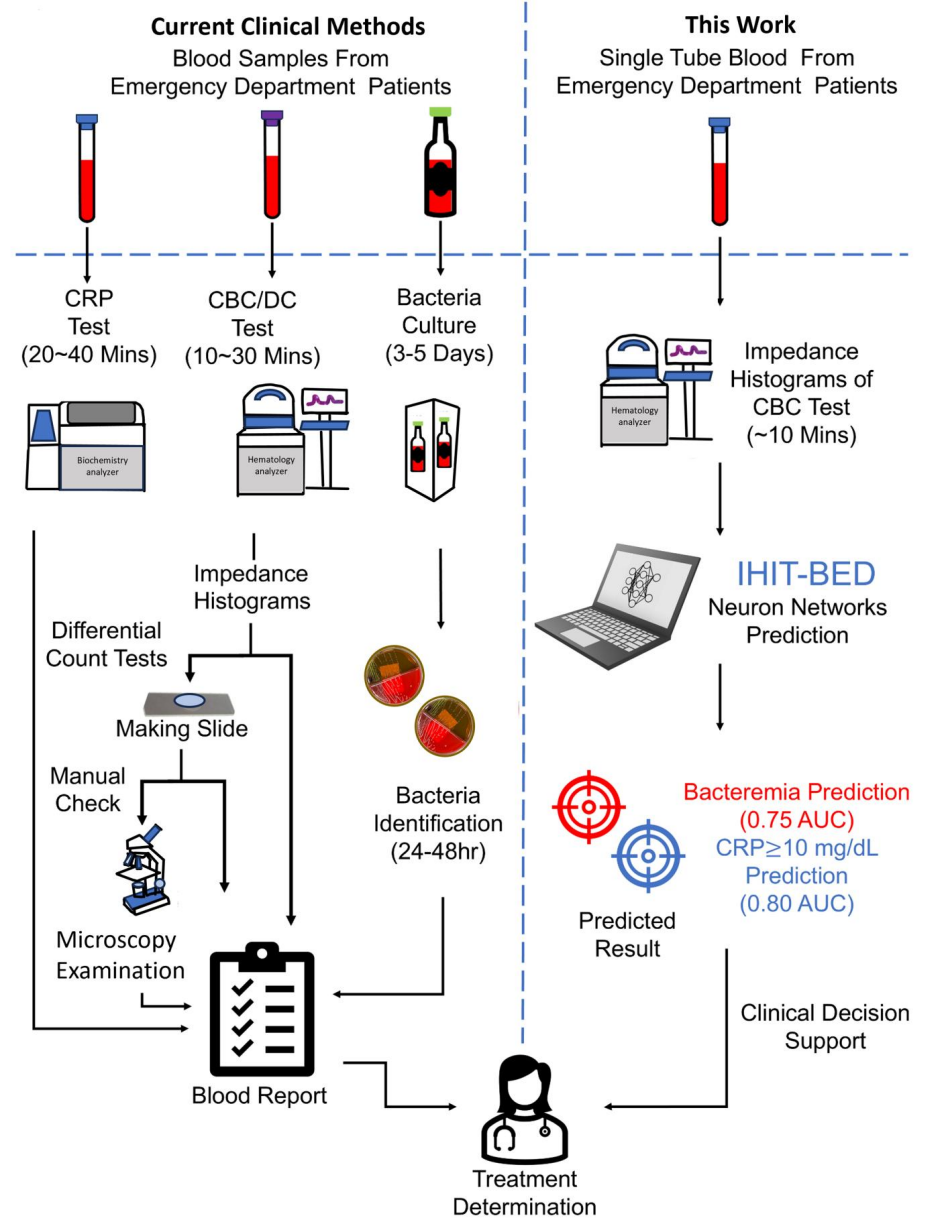
Comparison of diagnostic workflows between current clinical diagnostic methods and our proposed machine learning approach, Interpretable Hematology analyzer Impedance data-based Tabular network for early identification of Bacteremia in Emergency Department (IHIT-BED). Left: The standard workflow in the Emergency Department (ED), physicians order complete blood cell count (CBC), and differential count (DC) tests, C-reactive protein (CRP) test, and blood culture to evaluate the possibility of bacteremia or severe infection. In the clinical lab, these routine tests are time consuming. Among these, the blood culture process is time-consuming (typically >24 h). Right: In contrast, our proposed approach requires only the hematology impedance histograms of the CBC test, which is routinely performed in most ED patients. The impedance histograms generated by the standard hematology analyzer are directly extracted and used as input for the IHIT-BED model. The model predicts bacteremia and a high level of CRP in ∼10 min, which can further help with prompt clinical decision making for physicians in the ED. Part of the images were created using Solidworks version 2019 and Autodesk Inventor Sofware version 2023.

Therefore, in this study, we aimed to develop an interpretable transformer approach using unbiased hematology analyzer impedance data for early identification of bacteremia and severe inflammation in the emergency department (IHIT-BED). As shown in Fig. 1, the IHIT-BED can predict bacteremia and high levels of CRP in ∼10 min using impedance data from the first blood draw taken in the ED, which can further provide assistance for prompt clinical decision making for physicians.

## 2 Methods

**Figure 2.**
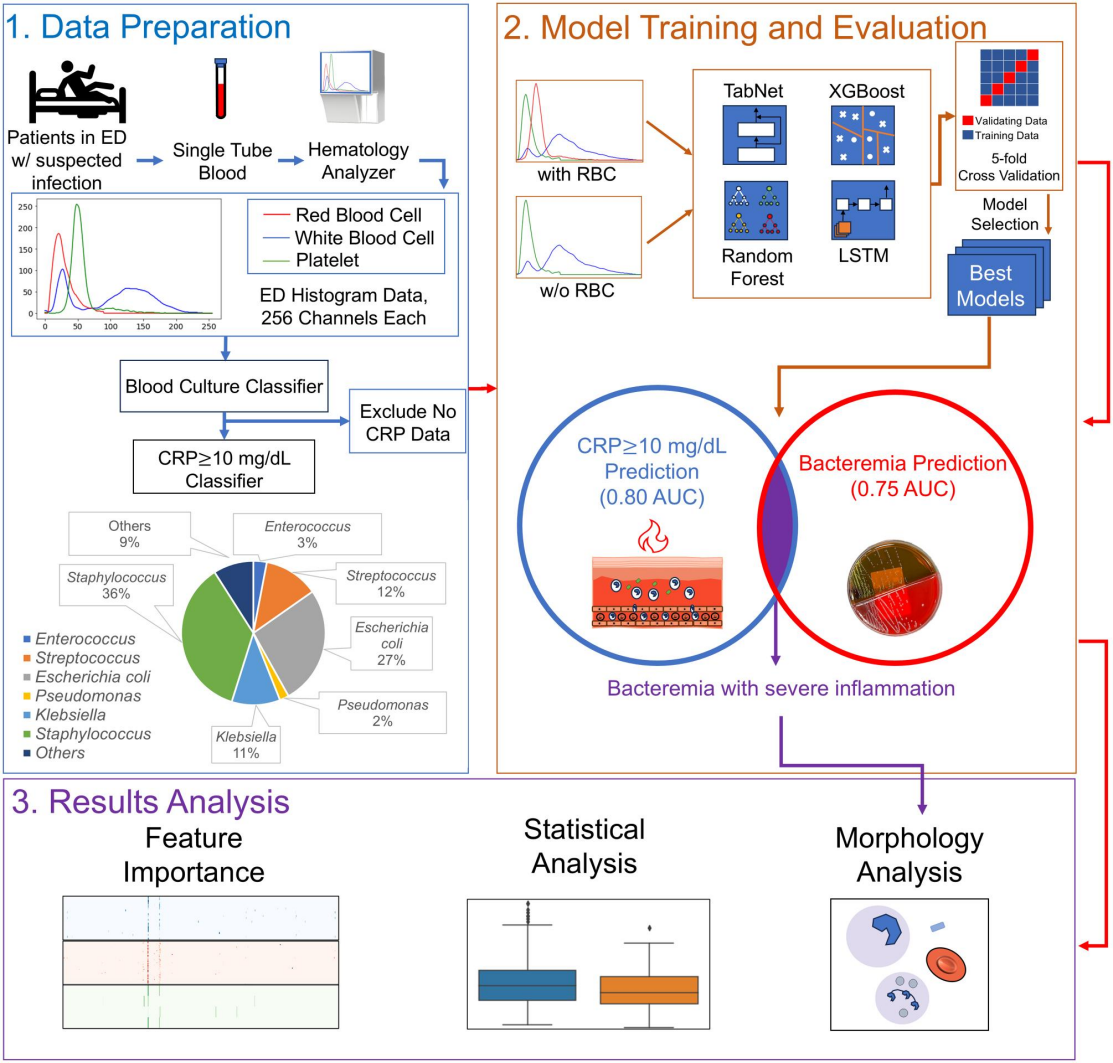
Schematic diagram of our experimental design. The experimental design consists of three diagrams: (1) Data preparation: Impedance histograms from blood samples of CBC tests ordered for patients visiting the ED with suspected infection were collected as input data for the blood culture classifier and the CRP classifier. (2) Model training and evaluation: Random Forest (RF), Extreme Gradient Boosting (XGBoost), Long Short Term Memory (LSTM), and Tabular Network (TabNet) models were selected, and the output performance of each model was evaluated; the two IHIT-BED classifiers (blood culture classifier and CRP classifier) using the TabNet model showed best performance in training and testing cohorts. (3) Results analysis: Feature importance analyses and TabNet heatmap analyses were performed, and compared between samples with combined positive blood culture and CRP ≥10 mg/dL results and samples without predicted combined positive results. Furthermore, the morphology analysis was performed, where we examined the differential counts and microscopic images in the second independent testing cohort to analyze the correlation between the predicted positive outcome of IHIT-BED and blood cell morphologies related to blood stream infection. Part of the images were created using Solidworks version 2019.

The experimental schema is shown in Fig. 2, consisting of (i) Data preparation, (ii) Model training and evaluation, and (iii) Result analysis. The detailed process of each step is described as below.

### 2.1 Data preparation

#### 2.1.1 Study population and data collection

We retrospectively collected impedance histogram data from blood samples of CBC tests ordered for patients visiting the ED of China Medical University Hsinchu Hospital (CMUHCH) during the following three periods: data collected between December 2022 and June 2023 were used as our training cohort; data collected from July 2023 were used as the first independent testing cohort, and data collected from February 2024 were used as the second independent testing cohort (Fig. 1, available as supplementary data at *Bioinformatics Advances* online). This study was approved by the Research Ethics Committee of the China Medical University Hospital (CMUH113-REC2-053).

Between December 2022 and June 2023, a total of 13 321 CBC tests were ordered in the ED, and the impedance data of these tests were collected as the training cohort (age 52.44±24.05 years, 49% male). To include only the impedance data derived from ED patients with clinical suspicion of blood stream infection, data without simultaneous blood culture test ordering (*n = *6962) were excluded for blood culture classifier training and data without simultaneous CRP tests (*n = *522) were further excluded for CRP classifier training. In the first independent testing cohort, impedance data of CBC tests derived from 1704 patients visiting ED were collected (age 55.05±23.79 years, 51% male), and underwent the same exclusion criteria as the training cohort. In the second independent testing cohort, impedance data derived from 181 CBC tests ordered in the ED were collected (age 51.23±22.35 years, 46% male).

#### 2.1.2 Class imbalance handling

Our data showed class imbalance: in the training cohort, the rate of positive outcome was 6.56% in the blood culture classifier, and was 19.39% in the CRP classifier. In the first independent testing cohort, the rate of positive outcome was 6.53% in the blood culture classifier, and was 17.89% in the CRP classifier. In the second independent testing cohort, the rate of positive outcome was 5.2% in the blood culture classifier, and was 18.18% in the CRP classifier. Since imbalance correction has been reported to confer mis-calibration reducing the clinical utility of prediction models (Van den Goorbergh *et al.* 2022) and was found to be at risk of introducing bias and compromising external validity (Halac *et al.* 2025), class imbalance correction was not applied in this study.

#### 2.1.3 Characteristics of hematology impedance histograms

Impedance data were generated using a Beckman Coulter DxH800 hematology analyzer. Briefly, the impedance signals were sensed by detection of transient current drop created by blood cells passing through the aperture of the hematology analyzer, which was proportional to the particle volume (Don). The impedance histogram values of WBC, PLT, and RBC were visualized on 256 channels, as shown in Fig. 2, available as supplementary data at *Bioinformatics Advances* online.

#### 2.1.4 Outcome definition

We defined the primary outcome as (i) positive blood culture (ii) positive blood culture accompanied with severe inflammation (detection of CRP level≥10 mg/dL).

### 2.2 Model training and evaluation

#### 2.2.1 Data preprocessing

This study used hematology impedance histogram data as the model input and CRP data and Blood Culture data as different model outputs. Because RBC parameters are not usually applied for clinical evaluation of blood stream infection (Sipahi *et al.* 2004, Singer *et al.* 2016), this study compared the training results with and without RBC histograms as inputs. To preserve the original features of impedance histograms, which are crucial for uncovering unknown knowledge, we refrained from using feature transformation methods, such as principal component analysis (PCA) (Bro and Smilde 2014) or data balancing (He and Garcia 2009).

#### 2.2.2 ML models for classification

This study implemented four models for predicting bacteremia using the 256 channel values of impedance signals acquired on the hematology analyzer: Random Forest (RF) (Breiman 2001), Extreme Gradient Boosting (XGBoost) (Chen and Guestrin 2016), Long Short Term Memory (LSTM) (Yu *et al.* 2019), and Tabular Network (TabNet) (Arik and Pfister 2021).

##### 2.2.2.1 *Model selection*

The RF and XGBoost models were selected based on previous reports on predicting bacteremia using tabular laboratory data (Li *et al.* 2022, Lien *et al.* 2022, Chang *et al.* 2023a). RF modeling generated multiple decision trees based on bagging of ensemble learning (Breiman 2001); while XGBoost generated multiple decision trees based on boosting of ensemble learning with the input tabular data of impedance histograms (Chen and Guestrin 2016). We implemented RF using the Python RandomForest library and XGBoost using the standard suite. RF aggregated multiple decision trees by majority vote, while XGBoost performed gradient boosting by optimizing a regularization objective at each split.

The LSTM model was selected to process the continuous signal data type of impedance histograms, using two hidden layer vectors, $h_{t}$ (short-term memory factor) and$\;C_{t}\;$(long-term memory factor). Furthermore, TabNet, which is a model using transformer architecture that has been suggested to process tabular data and sequence-like data (Khalili *et al.* 2022), was specifically selected as the machine learning model in this study. In TabNet model training, the architecture consisted of three major components: fully connected layer (FC), batch normalization (BN), and gate linear unit (GLU). The equations are described as below:


(1) FC: y=Wx+b

(2) $$\mathrm{BN}:\;\hat{y_{i}}=\frac{y_{i}-\mu_{i}}{\sqrt{{\sigma_{i}}^{2}+ɛ}}$$

(3) GLU: y=(Wx+b) ⊗ σ(Vx+c)

We used sequential attention modules “gate linear unit” (GLU) and interpretable masks to select features of impedance histograms for efficient and interpretable learning.

Detailed mathematical information on these models is provided in the Supplementary Methods, available as supplementary data at *Bioinformatics Advances* online.

#### 2.2.3 Hyperparameter tuning

Different models and classifiers have different optimized hyperparameters, which would directly affect the computing tendency of the model (Bergstra *et al.* 2011). This study used the grid searching method to auto-tune the best hyperparameters, and each hyperparameter would cut a maximum of 10 splits off. The tuning strategies of each model are described as follows, the details of the hyperparameters are shown in Table 1, available as supplementary data at *Bioinformatics Advances* online.

To prevent data imbalance-related overfitting in the RF model, we used limited tree split and tree depth as the hyperparameter tuning strategy. Similar strategy was applied to the tuning of T (the splits/nodes of the trees) and in the XGBoost model, with additional tuning of the regularized number, γ and λ. In LSTM, we tuned the hidden layer hyperparameters to optimize its performance in processing signal data as previously described (Yu *et al.* 2019). In TabNet, the number of the gate linear unit (GLU) and the number of the encoder, decoder hyperparameters were tuned as previously described (Arik and Pfister 2021).

Specifically, according to the learning curve of each hyperparameter in each model (Figs 6–9, available as supplementary data at *Bioinformatics Advances* online), we set n_estimator = 100, max_depth = 25 for classifiers in the RF model; n_estimator = 1200, γ = 1, λ = 1 for classifiers in the XGBoost model; learning rate = 0.005, hidden size = 256 for classifiers in the LSTM model; and learning rate = 0.001, n_step = 3, n_independent = 5 for classifiers in the TabNet model.

#### 2.2.4 Cross-validation

In this study, K-fold cross-validation (*K* = 5) was chosen to maximize the validation data and to minimize the training time (Fushiki 2011).

#### 2.2.5 Output model performance evaluation

A confusion matrix was created to evaluate the four following parameters: True positive (TP) indicates that both the classifier output and the laboratory test results detected positive blood culture or CRP ≥ 10 mg/dL; true negative (TN) indicates that neither the classifier nor the laboratory results showed positive blood culture/CRP ≥10 mg/dL; false positive (FP) indicates that the classifier incorrectly predicted a positive outcome but the laboratory test results were negative for blood culture or showing CRP < 10 mg/dL; false negative (FN) indicates the model predicted a negative outcome but the laboratory test results were positive for blood culture or showing CRP≥10 mg/dL.

This study used the area under the receiver operating characteristic (ROC) curve (AUC) as our evaluation standard for performance comparison among different classifiers and models (Chung *et al.* 2019). The ROC curve is a plot of the true-positive rate (TPR) versus the false-positive rate (FPR) of a diagnostic test. The AUC integrates the overall diagnostic test accuracy of the model. Unlike accuracy (ACC) only, AUC is more suitable to evaluate the model performance in this study with imbalanced datasets.

### 2.3 Results analysis

#### 2.3.1 Feature importance

Feature importance was evaluated to further interpret how the model correlated with the impedance histograms of the hematology analyzers. In the TabNet framework, importance scores were computed as masks across samples of the corresponding feature attributes. Then, we combined the importance scores on a heatmap to analyze the impact of the respective features on the overall model performance.

#### 2.3.2 Statistical analysis

This study compared whether the feature importance of CRP and blood culture classifier were similar. Moreover, *t* test and the B-H adjusted-*P* correction method were used to examine whether the features selected by each classifier could differentiate the respective outcome and the combined outcome (i.e. combine blood culture classifiers with CRP classifier).

#### 2.3.3 Morphology analysis

Based on the findings in Povoa *et al.* (1998), Koozi *et al.* (2020), and Jiang *et al.* (2023), this study combined the CRP and blood culture classifiers to predict bacteremia with severe inflammation. Fifteen samples in the second independent testing cohort were predicted to be positive in both the blood culture classifier and the CRP classifier. Among these 15 samples, only five had available blood smear (Fig. 1, available as supplementary data at *Bioinformatics Advances* online). Therefore, we further examined the DCs and microscopic images in these five samples to investigate the correlation between the predicted outcome of IHIT-BED and blood cell morphologies related to blood stream infection.

## 3 Results

### 3.1 IHIT-BED flow chart

We assessed the performance of the IHIT-BED with blood culture classifiers and CRP classifiers using cross-validation and two independent tests. The flow chart of the experiment is shown in Fig. 1, available as supplementary data at *Bioinformatics Advances* online.

### 3.2 Model performance of blood culture classifier in training cohort

**Figure 3.**
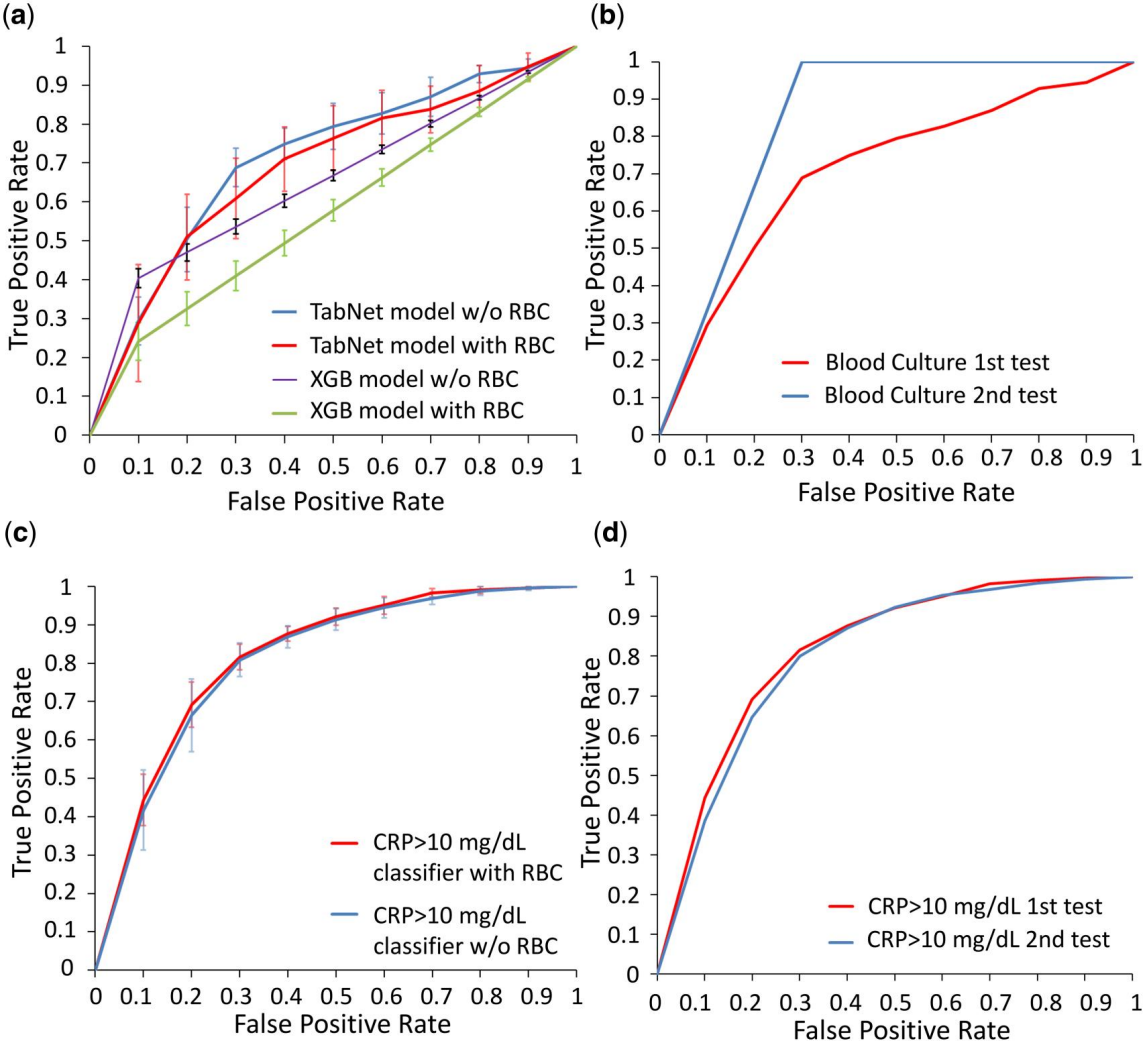
Model performance of blood culture and CRP classifiers in different cohorts. (a) The mean ROC curves of the XGBoost and TabNet models, with or without the RBC channel of the blood culture classifier in the training cohort of five-fold cross validations. Bars represent ±1 SD of the variation of the ROC curves. (b) The TabNet model was chosen for further blood culture classification in the two independent testing cohorts. Here presents the ROC curves of the two testing cohorts. (c) The mean ROC curves of TabNet models for CRP classification with five-fold cross validations are presented. Bars represent ±1 SD of the variation of the ROC curves. (d) The TabNet model was chosen for further CRP classification in the two independent testing cohorts. Here presents the ROC curves of the two testing cohorts.

In this study, we evaluated different inputs by using different models to test the best training variables for the blood culture classifier. Cross-validation was performed to confirm the quality of the models during training, and the model with the best validation performance was selected to predict the testing dataset. The cross-validation ROC curves of the XGBoost and TabNet models, with and without the RBC channel of the blood culture classifier, are shown in Fig. 3a. The blood culture classifier using the TabNet model without RBC data as the input achieved a better AUC (0.73± 0.02) than the AUC of the blood culture classifier with RBC data as the input (0.72 ± 0.04). The XGBoost model obtained lower AUCs (AUC = 0.69±0.03 without RBC input; AUC = 0.58± 0.02 with RBC input) than the TabNet model. The cross-validation AUCs of the LSTM (0.55± 0.07) and random forest (0.55 ± 0.07) were the lowest, as shown in Fig. 3, available as supplementary data at *Bioinformatics Advances* online.

These results suggest that RBC features provided no advantages and made the model more inconsistent during training in terms of average and standard deviation of AUC values. Furthermore, the AUC performance of the four models indicates that the transformer mechanism in TabNet outperformed conventional machine learning techniques in terms of mean AUC using hematology analyzer impedance histograms.

The AUC performance of the blood culture classifier in training and testing cohorts was shown in Table 1. To summarize, the blood culture classifier trained without RBC data using the TabNet model achieved the best training results, with a mean AUC of 0.73 and a standard deviation of 0.02. Therefore, we chose the TabNet model without RBC input for our classifier for testing.

**Table 1.** The AUC performance of the blood culture classifier in training and testing cohorts.^a^

	BC classifier training			
Model	TabNet w/o RBC	TabNet w/RBC	XGB w/o RBC	XGB w/RBC
Avg AUC	0.73	0.72	0.69	0.58
Std AUC	0.02	0.04	0.03	0.02

	BC classifier testing			
	
Model	TabNet w/o RBC			

Avg AUC	0.75			
Std AUC	0.85			

a AUC, area under the receiver operating characteristic (ROC) curve; Avg, average; BC, blood culture; Std, standard deviation; w/, with; w/o, without; XGB, XGBoost.

### 3.3 Performance of blood culture classifier in independent testing

To validate the accuracy of our evaluated blood culture classifier prediction further, two independent testing cohorts were used. The results of the first independent test of the blood culture classifier are shown in Fig. 3b. We had obtained an AUC of 0.72 using the blood culture classifier. In the first independent testing blood culture classifier confusion matrix, we noted that there were 31/79 false-negative samples. Further examination of the blood culture reports revealed that 16 of 31 false-negative samples were positive for Coagulase negative *Staphylococcus* (CoNS), which are common contaminants in blood cultures. We then re-labelled these 16 samples as blood culture negative, and obtained an AUC of 0.75 in our first independent testing cohort. In the second independent testing cohort, we achieved an AUC of 0.85 on the blood culture classifier.

These results suggest that the blood culture classifier using TabNet provides good performance in training and both the first and second independent testing cohorts. The Brier scores for the training cohort, first independent testing cohort, and second independent testing cohort were 0.19, 0.19, 0.17, respectively, suggesting these models were well-calibrated.

### 3.4 Model performance of CRP classifier in training cohort

Next, we evaluated different inputs using TabNet to test the best training variable of the CRP classifier. The cross-validation ROC curves of the model with and without the RBC channel of the CRP classifiers are shown in Fig. 3c. CRP classifier with RBC data as the input achieved a higher AUC (0.83± 0.02) than without RBC data as the input of the classifier (0.81± 0.02) (Table 2). In contrast to the blood culture classifier, this phenomenon indicates that RBC features have an indirect correlation with inflammation in terms of increased mean AUC. Therefore, we chose TabNet with RBC input as our testing model for CRP classifier.

**Table 2.** The AUC performance of the CRP classifier in training and testing cohorts.^a^

	CRP classifier training	
Model	TabNet w/o RBC	TabNet w/RBC
Avg AUC	0.81	0.83
Std AUC	0.02	0.02

	CRP classifier testing	
	
Model	TabNet w/RBC	

Avg AUC	0.83	
Std AUC	0.80	

a AUC, area under the receiver operating characteristic (ROC) curve; Avg, average; BC, blood culture; Std, standard deviation; w/, with; w/o, without; XGB, XGBoost.

### 3.5 Model performance of CRP classifier in independent testing

The first independent test results are shown in Fig. 3d. We achieved an AUC value of 0.83 for the CRP classifier. In the second independent testing cohort, we obtained the AUC value of 0.80 on CRP classifier (Table 2). The Brier scores for the training cohort, first independent testing cohort, and second independent testing cohort were 0.15, 0.17, 0.19, respectively, suggesting these models were well-calibrated.

Together, we established the IHIT-BED combining our best blood culture classifier and CRP classifier for bacteremia and severe inflammation prediction.

### 3.6 IHIT-BED prediction with combined classifiers in second independent testing

To further test the utility of IHIT-BED in the prognostication of bloodstream infection, we analyzed the performance of the combined blood culture and CRP classifier in predicting blood stream infection with severe inflammation. In the second independent testing cohort, we compared our predicted results with their clinical test reports. In 15 IHIT-BED predicted positive cases in both blood culture classifier and CRP classifier, three patients had positive blood culture reports, and eight patients had CRP≥10 mg/dL report. Therefore, 73.33% of IHIT-BED predicted positive cases are in need of immediate antibiotic care.

To this end, we developed the IHIT-BED to rapidly identify blood stream infections and severe inflammation, which can be applied to assist in the clinical evaluation of sepsis and prompt antibiotics treatment.

### 3.7 Feature importance of blood culture classifier and CRP classifier

**Figure 4.**
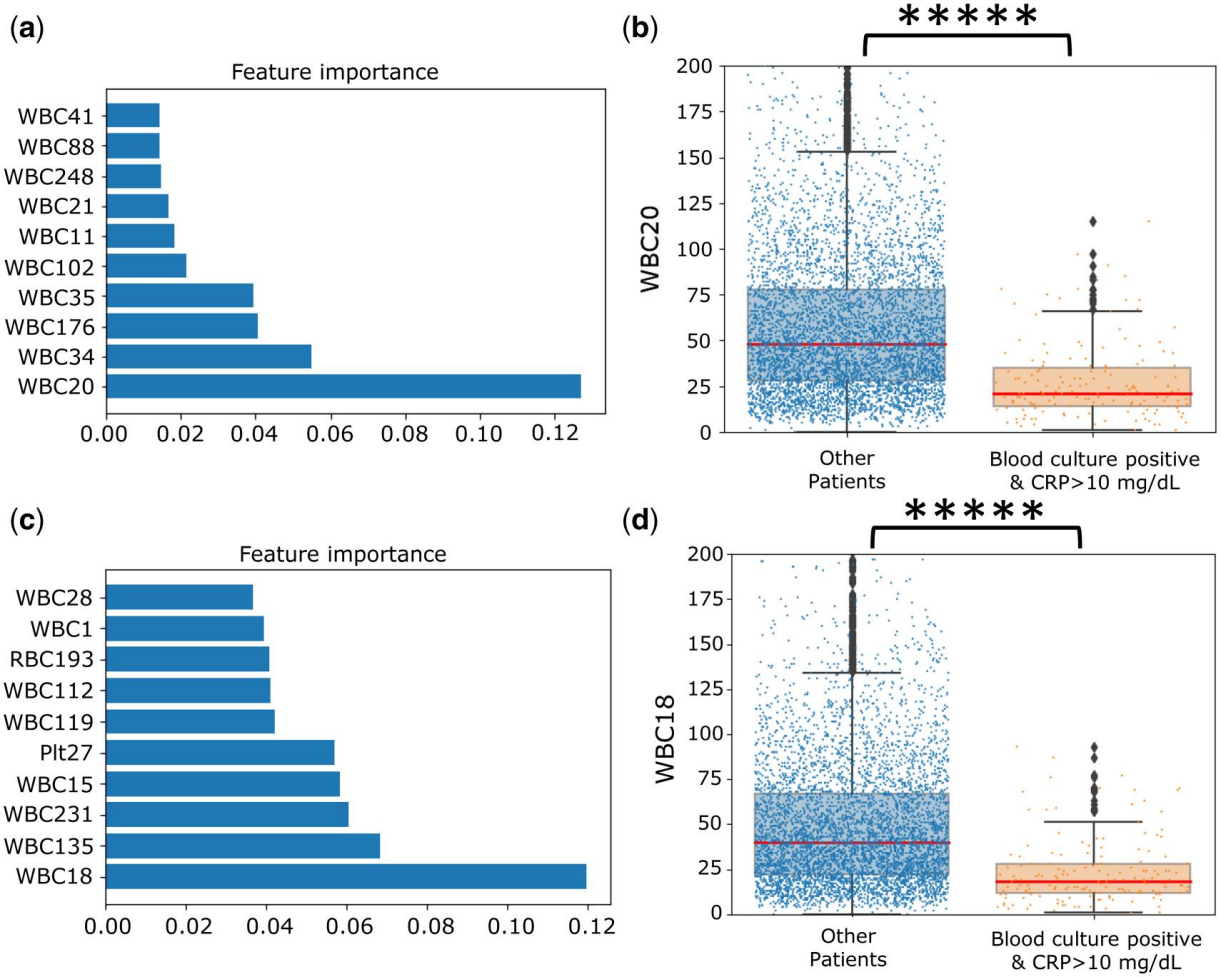
Feature importance of the blood culture classifier and of the CRP classifier in the training cohort. (a) Top 10 feature importance of the blood culture classifier. (b) Box plots of the WBC20 histogram values of samples from the combined predicted positive outcome (the group of blood culture + and CRP ≥ 10 mg/dL, *n = *160) and from the group with other outcomes (*n = *5266). *****P < 10^−10^ calculated by t test with B-H correction (c) Top 10 feature importance of the CRP classifier. (d) Box plots of the WBC18 histogram values of samples from the combined predicted positive outcome (the group of blood culture + and CRP≥ 10 mg/dL, *n = *160) and from the group with other outcomes (*n = *5266). *****P < 10^−10^ calculated by *t* test with B-H correction.

To further interpret how the model correlated with the hematology analyzers’ impedance histograms of the training cohort, we performed feature importance analyses. The top 10 feature importance values of the blood classifier were WBC 11, 20, 21, 34, 35, 41, 88, 102, 176, and 248, as shown in Fig. 4a. The top 10 feature importance values of the CRP classifiers were WBC 1, 15, 18, 28, 112, 119, 135, 231, PLT 27, and RBC 193, as shown in Fig. 4c. From these results, we found that several of the importance chosen from the different classifiers have similar sizes on the impedance histogram, such as WBC 20 of the blood culture classifier and WBC 18 of the CRP classifier, and WBC 102 of the blood culture classifier and WBC 112 of the CRP classifier.

Furthermore, WBC 20 is the most important feature in blood culture classifier, and WBC 18 is the most important feature in CRP classifier. Since positive blood culture and CRP≥10 mg/dL would suggest severe blood stream infection, we further examined whether the selected feature could differentiate between the patients with combined positive clinical reports (blood culture positive report and CRP≥10 mg/dL report) and patients without combined positive clinical reports using T-tests. We found that WBC 20 and WBC 18 can differentiate between the two groups (both BH adjusted *P* value <10^−10^), as shown in Fig. 4b and d. These results revealed that WBC 20 and WBC 18 identified from blood culture classifier and CRP classifier can predict the combined positive blood reports, suggesting blood stream infection and severe inflammation.

### 3.8 TabNet heatmap analyses of blood culture classifier and CRP classifier

We further investigated whether feature importance other than the top 10 features mentioned above could also identify the severe blood stream infection group by extracting the TabNet heatmap (256 WBC features and 256 PLT features in blood culture classifier; 256 WBC features, 256 RBC features and 256 PLT features in CRP classifier) of the second independent testing. We compared features between samples with combined positive blood culture and CRP ≥ 10 mg/dL results and samples without predicted combined positive results.

**Figure 5.**
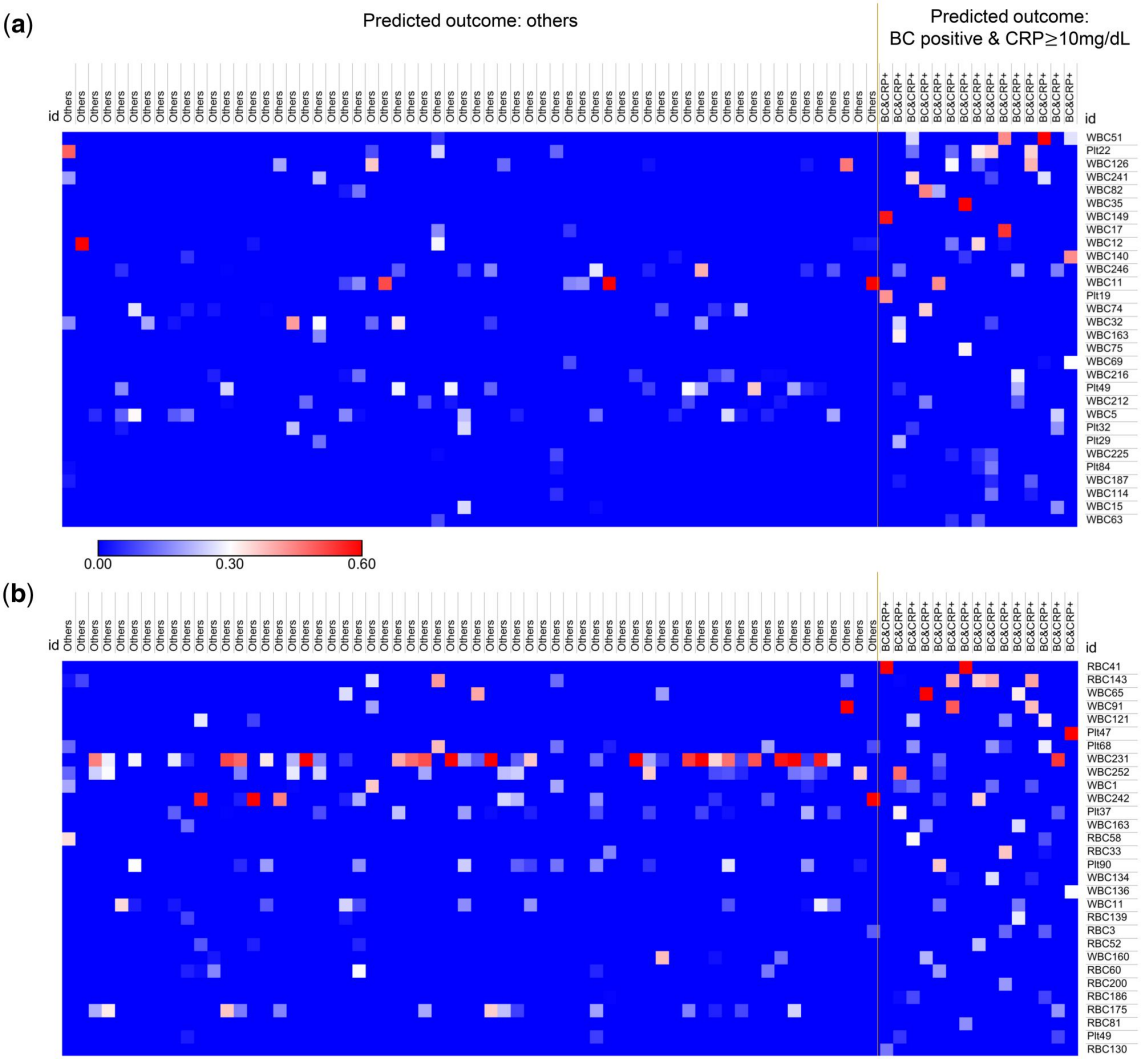
Feature importance of the TabNet heatmaps visualized by ranking the average value of every feature mask in the group of samples with predicted combined positive outcome (blood culture positive and CRP ≥ 10 mg/dL). The top 30 features used by the TabNet model to predict the combined positive outcome using blood culture classifier (a) or CRP classifier (b) are shown. The color scheme represents heatmap values from 0 to 1. “Predicted outcome others” group, *n = *62; “Blood culture (BC) positive and CRP ≥10 mg/dL” group, *n = *15. These heatmaps were analyzed using Morpheus (https://software.broadinstitute.org/morpheus/).

The heatmaps were visualized by ranking the average value of every feature mask in the group of samples with predicted combined positive outcome (blood culture + and CRP ≥ 10 mg/dL). Three TabNet heatmaps were generated, however, only the first heatmap showed features potentially differentiating the two groups. The top 30 features in the first heatmap of each classifier are shown in Fig. 5a and b. Among the 512 features in blood culture classifier, we identified the WBC51, PLT22, and WBC126 as the top three additional features potentially differentiating the two groups as shown in Fig. 5a. Also, we identified RBC 41, RBC 143, and WBC65 as the top three additional features in the CRP classifier, as shown in Fig. 5b. The complete TabNet feature masks are shown in Figs 4 and 5, available as supplementary data at *Bioinformatics Advances* online. To summarize, we identified additional features by the heatmap analysis, which might be correlated with specific blood smear morphology.

### 3.9 Blood smear examination with feature importance and blood smear morphology

**Figure 6.**
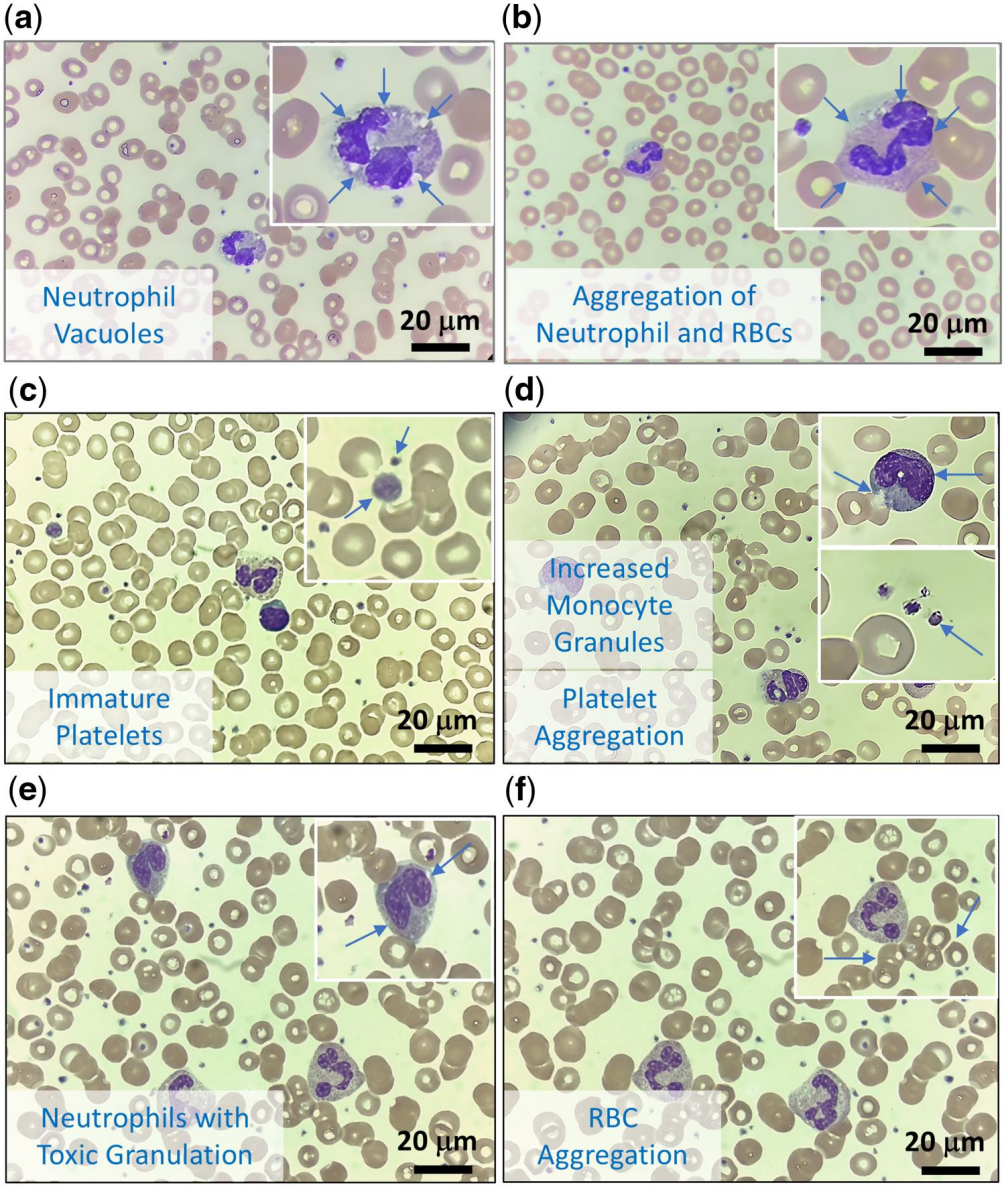
Representative blood smear images in samples of the predicted combined positive outcomes in the second independent testing cohort. (a) Neutrophil vacuoles. (b) Aggregation of neutrophil and red blood cells. (c) Immature platelets. (d) Increased monocyte granules and platelet aggregation. (e) Neutrophil with toxic granulation. (f) Aggregation of red blood cells. (e and f are from the same patient.)

To further examine the correlation between feature importance and blood smear morphology, we examined five blood smear slide samples in the second independent testing cohort. These images are shown in Fig. 6. We observed toxic granules, monocyte vacuoles, aggregation of neutrophils, red blood cells, and platelets, aggregation of neutrophils and red blood cells, immature platelets, increased monocyte vacuoles and platelet aggregation, neutrophil activation, and aggregation of red blood cells, consistent with the morphological signs of infections.

Since impedance histogram features (256 channels, as shown in Fig. 2, available as supplementary data at *Bioinformatics Advances* online) were proportional to the volume/size of blood cells, and the value of each feature correlated to the cell count/peak of histogram, we aligned the identified important features to the distribution curve of WBC (lymphocytes, monocytes and neutrophils were further differentiated by different positions), platelet and RBC histograms as described previously (Thomas *et al.* 2017b). Of note, we found that the top three model heat map-derived features, PLT22, WBC51, WBC126, in our blood culture classifier might correspond to the peaks of thrombocytosis, activated lymphocytes, activated monocytes, left shift and aggregation of neutrophils and RBCs in CBC impedance histograms. Also, the top three model heat map-derived features, RBC41, RBC143 and WBC65 in our CRP classifier might correspond to the peaks of immature/giant platelet, the aggregation of RBC, and activated monocytes.

These results show that the features identified by our blood culture classifier and the CRP classifier capture the morphological changes in blood stream infection. In addition, these features might also detect blood smear morphologies that are not routinely examined in clinical labs, such as increased neutrophil granules and the aggregation of cells.

## 4 Discussion and conclusion

In this study, we established the first hematology impedance histogram-based prediction system, IHIT-BED, to evaluate the risk and severity of bloodstream infection in patients visiting the ED in ∼10 min. To the best of our knowledge, the IHIT-BED is the first predictor based on hematology impedance histogram signals. The IHIT-BED using TabNet provides good performance in training and in the first and second independent testing. Our data suggest that IHIT-BED can detect features indicative of bacteremia and inflammatory immune responses. We showed that the application of the IHIT-BED can identify severe blood stream infection rapidly using the first blood draw in the ED, which can alert physicians for prompt treatment initiation.

Different clinical deep learning models have been developed to assist early detection of diseases (Yu *et al.* 2019, Tsuneki 2022). While convolutional neural network (CNN)-based models were commonly selected for training using the image data (Abrantes 2023, Diogo *et al.* 2023) transformer-based and tree-based deep learning models were applied to establish predictors for bacteremia using discrete laboratory data, such as CBC counts, DC and cell population data (Lien *et al.* 2022, Chang *et al.* 2023a, Xia *et al.* 2024), and combined multi-model approaches have been suggested to build predictors for blood stream infection (BSI) using laboratory and temporal data extracted from EHR (Hirszowicz and Aran 2024, Amrollahi *et al.* 2025). The main challenge in studies using multiple laboratory data to predict BSI is the large number of missing values, which impacted dataset integrity and statistical reliability. Furthermore, preprocessing of missing values with imputation techniques could predispose to data drifting (Zhang *et al.* 2008, Alam *et al.* 2023). For example, Chang *et al.* developed ML model to predict bacteremia in patients visiting ED with a good AUC of around 0.84, however, they replaced the missing values in DC with zeroes, and imputed the missing values in other blood tests and CPDs using median values of the training dataset, which might affect data validity and limit its clinical utility (Chang *et al.* 2023a). IHIT-BED is unique in that we used unbiased hematology impedance histograms as input, which are channel tabular values derived from continuous detection of signals of current change using the Coulter principle. Unlike the CPD data which could only be acquired in the more advanced hematology analyzer, the impedance histograms are widely available on routine analyzers. The hematology impedance histograms had no missing values, thus overcoming the concern of data drifting in the model performance on new data. Moreover, IHIT-BED preserved the original features of the impedance signals without any feature preprocessing, which allowed us to discover potential novel histogram-related parameters for predicting bloodstream infection and inflammation. The turnaround time of the IHI-BED is also much shorter than ML models using the DC test, which sometimes required manual microscopic examination of blood smears.

In our study, IHIT-BED using TabNet, a transformer-based attentive interpretable tabular learning model, to predict positive blood cultures based on 768 features (256 each in WBC, PLT, and RBC channels) of hematology impedance histograms acquired upon ED visit achieved the AUC of 0.75 and 0.85 in the two independent testing cohorts, respectively. We found that the transformer based TabNet model had the best performance as compared with the tree-based models and LSTM in managing the impedance histograms, which exhibited similar data type to continuous signals. Furthermore, feature masks of TabNet offered insights in correlations of features of impedance histograms with blood cell morphologies potentially related to early signs of BSI. In our analysis of the correlation between positive IHIT-BED prediction and blood smear morphology, we observed not only neutrophil-predominant patterns, but also new parameters suggesting bacteria-related immune responses, such as atypical neutrophils (active cells with enlarged vacuoles, neutrophils that form clusters with RBCs, and neutrophils that form clusters with platelets) (Mauler *et al.* 2016, Lizcano *et al.* 2017). In addition, feature importance in the blood culture classifier, such as PLT22, WBC51, and WBC126; and feature importance in the CRP classifier, such as RBC41, RBC143, and WBC65, correspond to blood smear morphologies of activated platelet and leukocytes, and aggregation of platelets, which are morphologies potentially related to bacterial infections. Moreover, these features also suggest the presence of acute infection-related thrombocytosis (Schattner *et al.* 2019), neutrophil left shift/neutrophil morphological changes in infection (Seebach *et al.* 1997, Honda *et al.* 2016), immature/giant platelets which could be associated with severe bacteremia (Thorup *et al.* 2020), and activated monocytes having larger size or increased MDW potentially reactive to sepsis (Crouser *et al.* 2019). These results are consistent with previous reports that impedance histograms might provide further information about infection (Nishimura *et al.* 2021) and inflammation (Thomas *et al.* 2017a). Detection of outlier or abnormal individual peak of the CBC histogram as flag on the hematology analyzer has been suggested to alert clinicians for further morphological examinations (Dixit *et al.* 2022). However, the clinical utility of multiple flags was limited due to the requirement of expertise to interpretate these data. We have shown that IHIT-BED is able to integrate the impedance histogram signals promptly and predicts BSI. Therefore, IHIT-BED could be applied as a middleware of the hematology analyzer, collecting CBC histogram data and running the TabNet model in real time. Subsequently, IHIT-BED-predicted positive samples with high heatmap signals of important features (PLT22, WBC51, WBC126, RBC41, RBC143, and WBC65) could be flagged for further blood smear examination and blood culture testing. Interestingly, IHIT-BED classified the impedance histograms of a subset of samples with positive blood culture of common skin containments as negative for BSI, which hinted that the features of these impedance histograms might be different from those of pathogenic bacteremia. Further study analyzing more impedance histograms derived from patients having blood culture tests positive for pathogenic bacteria or skin contaminants, CoNS, using IHIT-BED algorithms would enable the validation of the potential of our model to differentiate pathogenic from non-pathogenic blood culture results.

Of note, there are limitations in this study. It has been reported that small-size dataset might contribute to overfitting, and the generalizability of the model could be hindered without external validations (Stylianides *et al.* 2025). IHIT-BED was built on retrospective cohorts derived from a single center; therefore, bias could be introduced in deep learning. A prospective study of larger size using impedance histograms derived from first blood test of patients with suspected infections visiting ER and with multi-center validation cohorts is needed to further test the generalizability and real-world clinical utility of IHIT-BED.

In conclusion, we have established a novel hematology impedance histogram-based prediction system, IHIT-BED, which could be applied in common routine hematology laboratories. With further validation studies, IHIT-BED might assist the rapid detection of bacteremia and severe inflammation using the first blood draw in the ED.

## Supplementary Material

vbaf322_Supplementary_Data

## Data Availability

The scripts and datasets used for the development of IHIT-BED in this study are available at the website: https://github.com/appleRtsan/IHIT-BED.
