# Supplementary material for: IHIT-BED: an interpretable transformer approach using unbiased hematology analyzer impedance data for early identification of bacteremia in emergency department
Source: Bioinform Adv. 2025 Dec 23;6(1):vbaf322. doi: 10.1093/bioadv/vbaf322 (PMC12895069; doi:10.1093/bioadv/vbaf322)
Supplement: vbaf322_Supplementary_Data [file vbaf322_supplementary_data.docx]

**IHIT-BED: An interpretable transformer approach using unbiased hematology analyzer impedance data for early identification of bacteremia in emergency department**

**Table of contents**

Supplementary Methods……………….. p.2-5

Supplementary Tables………………….. p. 6

Supplementary Figures………………… p. 7-14

**Supplementary Methods**

**Random Forest**

Random forest (RF) is a sort of ensemble model that involves the aggregation of multiple decision tree classifiers. Based on the integration of multiple decision trees within a RF model, each tree was generated from a subset of k attributes randomly selecting from training dataset with a total of m attributes, where k is less than m. In this way, we can obtain multiple decision-making results. Typically, the majority voting method is adopted to integrate the results to make a final decision, based on the class label with the most votes. The performance of a RF decision-making system is associated with the dimension of random vector, which is the number of attributes (k) used in each decision tree [1]. In this study, “Random Forest” package which constructed in python was utilized to construct RF classifiers based on various attribute sets.

**XGBOOST**

In every split, XGBoost would calculate the objective function below:

|  | $Objective= \sum_{i=1}^{n} loss(y_{i},\hat{y_{i}})+\gamma T+\frac{1}{2}\lambda\sum_{j=1}^{T} {w_{j}}^{2}$ |  |
| --- | --- | --- |

Where $n$ is the amount of data, $y_{i}$ is true label, $\hat{y_{i}}$ is predicted label, $T$ is the splits (nodes) of the trees and $w_{j}$ is its weight, $\gamma$ and $\lambda$ are the regularized number, $loss$ is the loss function. With the value of the loss function minimized, the model would exhibit its optimal performance, showcasing the best results.

**Long Short-Term Memory**

Long Short-Term Memory (LSTM) is a sort of recurrent neuron networks (RNN) [2], which is a neural network that processes sequence input and can process input data of variable length, such as sounds, videos, or articles. At the same time, its output can also be of variable length, and it all depends on the network to decide when to stop output. The characteristic of RNN is that it will design a hidden vector that cannot be learned but will be transmitted in the data sequence. We call it a hidden vector, which is used to help RNN use past information processing to predict future results. The LSTM and RNU mentioned later belong to one of the subcategories of RNN.


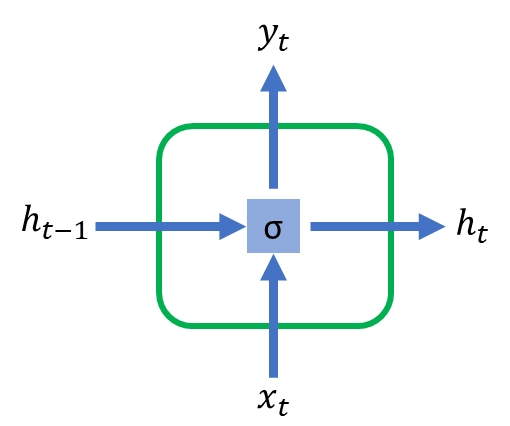


*Structure of the hidden layer of RNN*

In the graph above, we have the input layer $x_{t}\boldsymbol{\in}\mathbb{R}^{m*1}$ and hidden layer vector $h_{t}\boldsymbol{\in}\mathbb{R}^{n*1}$, where t $\boldsymbol{\in}\mathbb{R}$is the total amount of the hidden layer. The $\boldsymbol{U}_{\boldsymbol{h}}\boldsymbol{\in}\mathbb{R}^{n*m}$and the $\boldsymbol{V}_{\boldsymbol{h}}\boldsymbol{\in}\mathbb{R}^{n*n}$ are the parameters metrices. The output layer $y_{t}\boldsymbol{\in}\mathbb{R}^{n*1}$ is determined by$W_{y}$and $h_{t}$. The feed forward function can be determined as follows:

|  | $\boldsymbol{h}_{\boldsymbol{t}}\boldsymbol{=}\boldsymbol{\sigma}_{\boldsymbol{h}}\boldsymbol{(}\boldsymbol{U}_{\boldsymbol{h}}\boldsymbol{x}_{\boldsymbol{t}}\boldsymbol{+}\boldsymbol{V}_{\boldsymbol{h}}\boldsymbol{h}_{\boldsymbol{t-1}}\boldsymbol{+}\boldsymbol{b}_{\boldsymbol{h}}\boldsymbol{)}$ |  |
| --- | --- | --- |
|  | $\boldsymbol{o}_{\boldsymbol{t}}\boldsymbol{=}\boldsymbol{\sigma}_{\boldsymbol{y}}\boldsymbol{(}\boldsymbol{W}_{\boldsymbol{y}}\boldsymbol{h}_{\boldsymbol{t}}\boldsymbol{+}\boldsymbol{b}_{\boldsymbol{h}}\boldsymbol{)}$ |  |


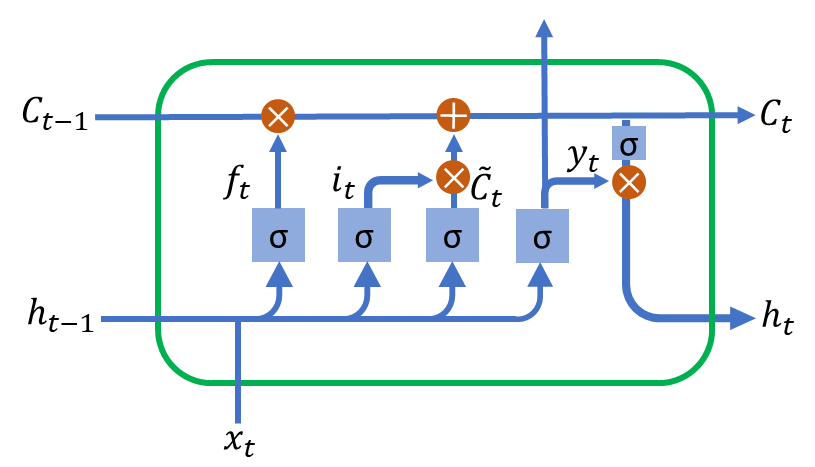


*Structure of the hidden layer of LSTM*

Similar structure to RNN, LSTM uses two hidden layer vector $h_{t}$ and $C_{t}$, where $h_{t}$ is the short-term memory factor and $C_{t}$ is the long-term memory factor. The feed forward function can be determined as follows:

|  | $\boldsymbol{f}_{\boldsymbol{t}}\boldsymbol{=\sigma(}\boldsymbol{W}_{\boldsymbol{f}}\boldsymbol{\cdot[}\boldsymbol{h}_{\boldsymbol{t-1}}\boldsymbol{,}\boldsymbol{x}_{\boldsymbol{t}}\boldsymbol{]+}\boldsymbol{b}_{\boldsymbol{h}}\boldsymbol{)}$ |  |
| --- | --- | --- |
|  | $\boldsymbol{y}_{\boldsymbol{t}}\boldsymbol{=}\boldsymbol{\sigma}_{\boldsymbol{y}}\boldsymbol{(}\boldsymbol{W}_{\boldsymbol{y}}\boldsymbol{h}_{\boldsymbol{t}}\boldsymbol{+}\boldsymbol{b}_{\boldsymbol{h}}\boldsymbol{)}$ |  |

**TabNet**

The transformers consist of three major components: fully connect layer (FC), batch normalization (BN), and gate linear unit (GLU). The equations were described below:

|  | $\mathrm{FC}\boldsymbol{: y=Wx+b}$ |  |
| --- | --- | --- |
|  | $BN: \hat{y_{i}}\mathbf{=}\frac{y_{i}-\mu_{i}}{\sqrt{{\sigma_{i}}^{2}+\epsilon}}$ |  |
|  | $GLU:\boldsymbol{y}\mathbf{=}\left( \boldsymbol{Wx}\mathbf{+}\boldsymbol{b} \right)\boldsymbol{\bigotimes\sigma(}\boldsymbol{V}\boldsymbol{x}\mathbf{+}\boldsymbol{c}\mathbf{)}$ |  |

FC consists of vectors and matrixes. Where $\boldsymbol{y}$ $\boldsymbol{\in}\mathbb{R}^{n}$is the output (hidden) vector, $\boldsymbol{x}$ $\boldsymbol{\in}\mathbb{R}^{m}$is the input vector, $\boldsymbol{W}\mathrm{and}\boldsymbol{V}\boldsymbol{\in}\mathbb{R}^{m*n}$ are the weight matrices, and $\mathbf{b}$ $\boldsymbol{\in}\mathbb{R}^{\boldsymbol{n}}$is the bias vector. After FC, there is BN layer to normalize each element. Where $\boldsymbol{y}_{\boldsymbol{i}}$ is $\boldsymbol{i}^{\boldsymbol{th}}$ element of output $\mathbf{y}$. $\mu_{i}$, $\sigma_{i}$ are respectively the value of the mean and standard deviation of this neuron, and $\epsilon$ is the number to prevent $\sigma_{i}$ is zero. GLU is an activation function, where $\boldsymbol{\sigma}$ is sigmoid function and ⊗ is the element-wise product between matrices.

After processing the features using attentive transformer, the model split for the decision step output $\boldsymbol{d}_{\boldsymbol{out}}$. The model Apply a linear mapping $\boldsymbol{W}_{\boldsymbol{final}}\boldsymbol{d}_{\boldsymbol{out}}$ to get the outcome.

**Reference**

[1] Belgiu, M., & Drăguţ, L. (2016). Random forest in remote sensing: A review of applications and future directions. *ISPRS journal of photogrammetry and remote sensing*, *114*, 24-31.

[2] Sherstinsky, A. (2020). Fundamentals of recurrent neural network (RNN) and long short-term memory (LSTM) network. *Physica D: Nonlinear Phenomena, 404*, 132306.

**Supplementary Tables**

Supplementary Table S1. Hyperparameters of each model

| **Model name** | **Hyperparameter** | **Hyperparameter value range** |
| --- | --- | --- |
| Random Forest | N_estimator | 100,200,400,800,  1200,1500,2000 |
|  | Max_depth | 2,5,8,12,15,  20,25,30,40,50 |
| XGBoost | N_estimator | 100,200,400,800,  1200,1500,2000 |
|  | Lambda | 1,2,3,4,5,6,7,8,9,10 |
|  | Gamma | 1,2,3,4,5,6,7,8,9,10 |
| TabNet | Learning rate | 0.001,0.005,0.008  0.01,0.02,005 |
|  | N_independent (GLU) | 1,2,3,4,5 |
|  | N_steps  (Number of Encoder & Decoder) | 1,2,3,4,5 |
| LSTM | Learning rate | 0.001,0.005,0.008  0.01,0.02,005 |
|  | Hidden size | 256,512,768 |

**Supplementary Figures**


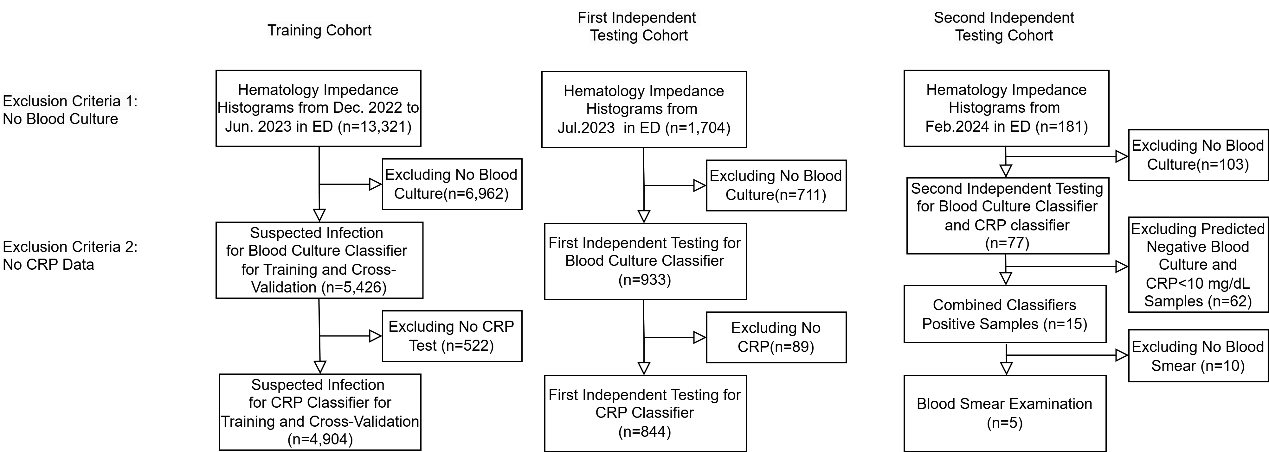


**Supplementary Figure S1.** Experimental flow charts

.


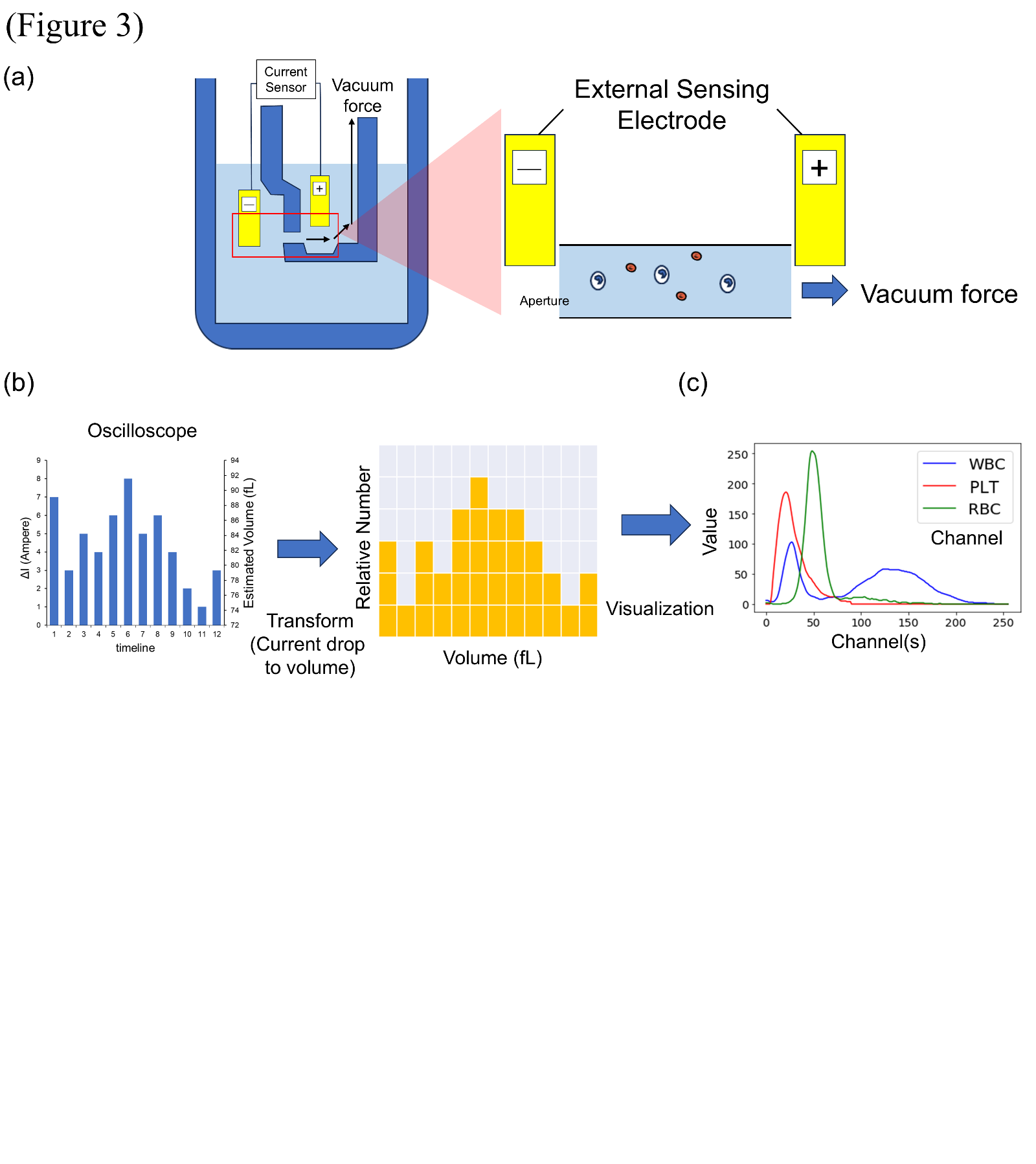


**Supplementary Figure S2.** Acquisition of hematology impedance histograms. (a) The impedance signals were sensed by detection of transient current drop created by blood cells passing through the aperture of the hematology analyzer. (b) The current drop was proportional to the volume of blood cells. (c) The impedance histogram values of WBC, PLT and RBC were visualized on 256 channels.


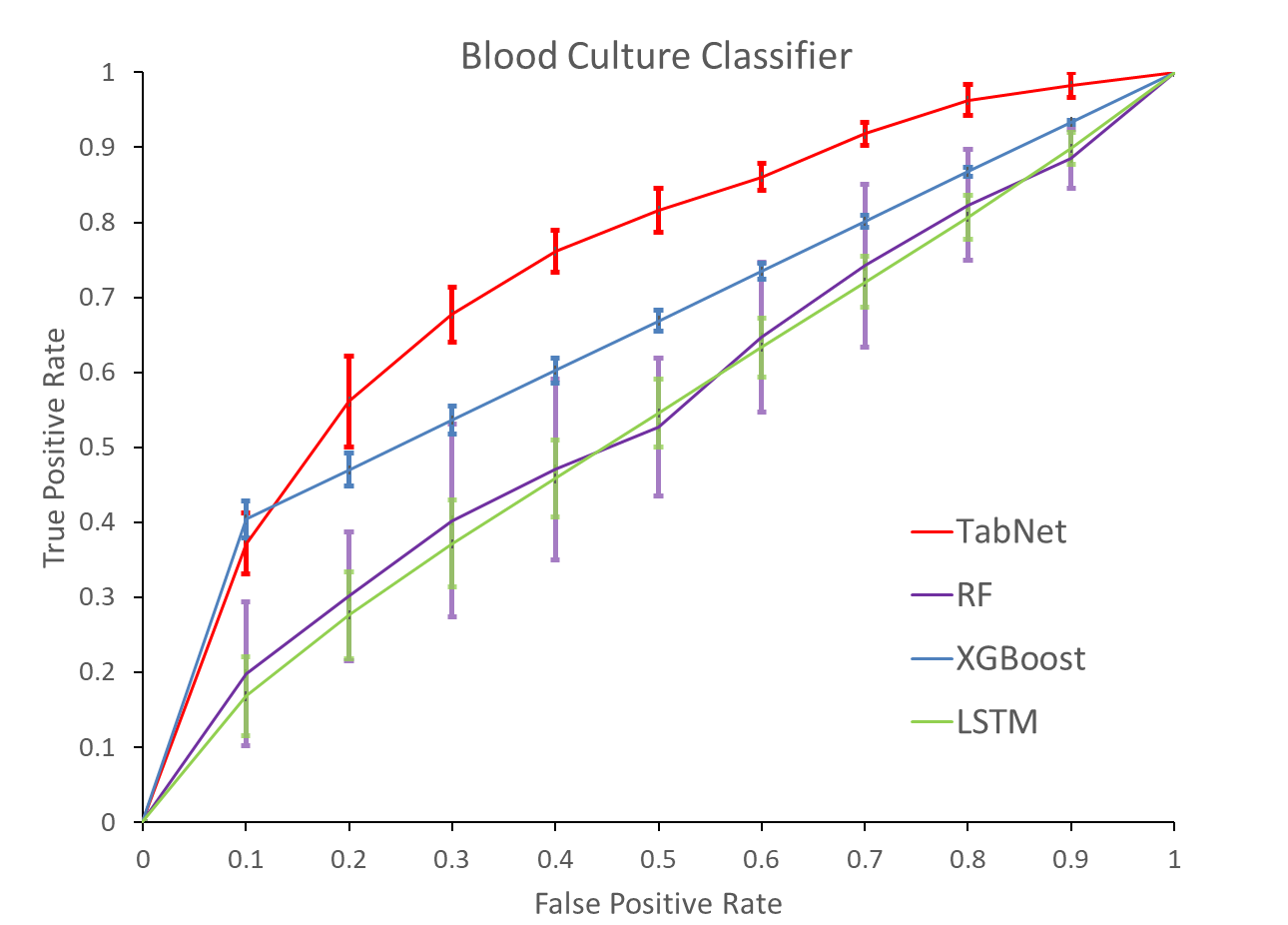


**Supplementary Figure S3**. ROC curves of 4 different models of blood culture classifier. The mean ROC curves of the four models without the RBC channel of the blood culture classifier in the training cohort of five-fold cross validations. Bars represent ± 1SD of the variation of the ROC curves.


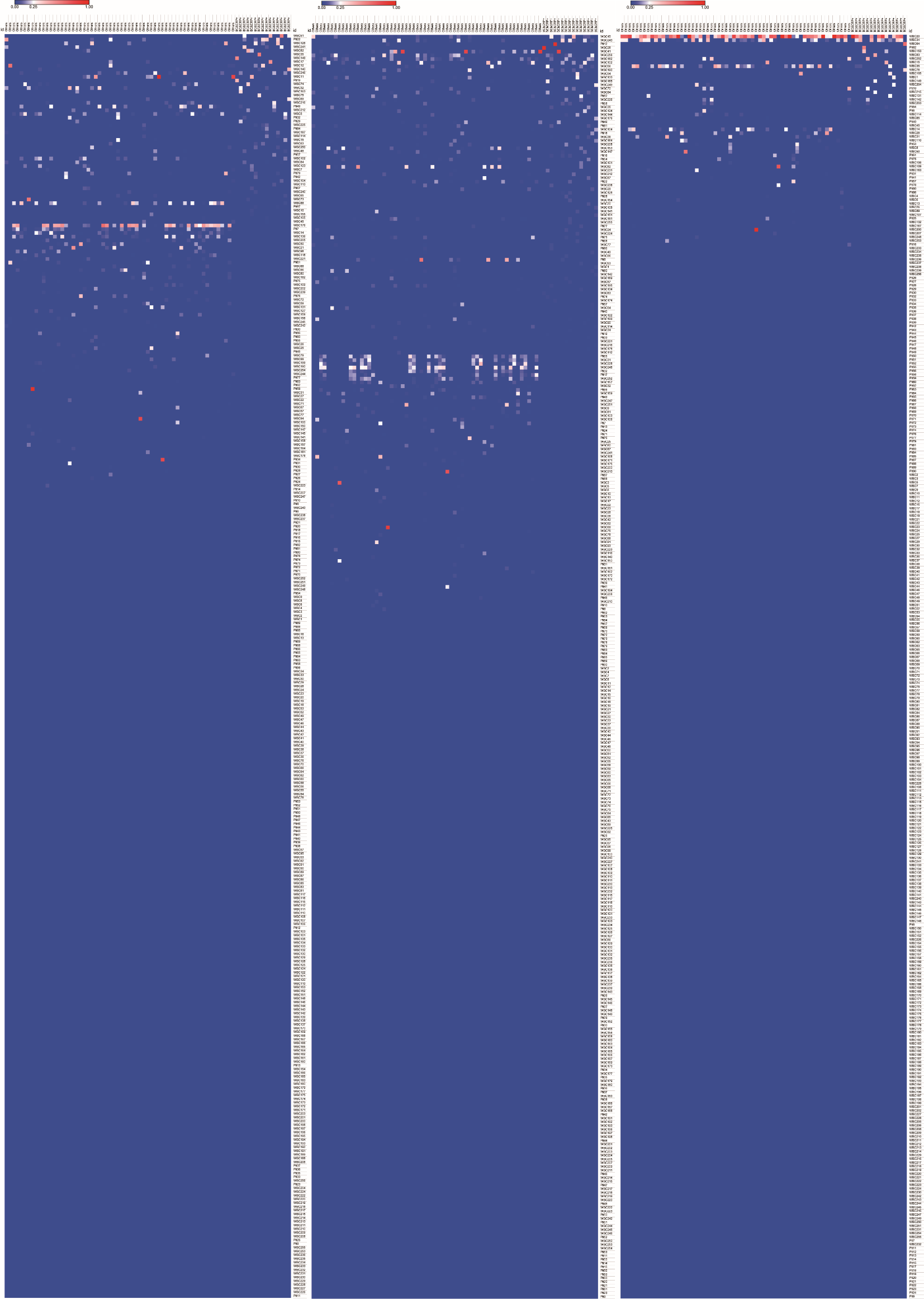


**Supplementary Figure S4.** The all features in the all three heatmaps of the blood culture classifier. The color scheme represents heatmap values from 0 to 1. “Predicted outcome others” group, n=62; “Blood culture (BC) positive and CRP ≥10 mg/dL” group, n=15. Left: The first heatmap, which is the least near layer to the BC output. Middle: The second heatmap, which is the second near layer to the BC output., Right: The third heatmap, which is the nearest layer to the BC output.


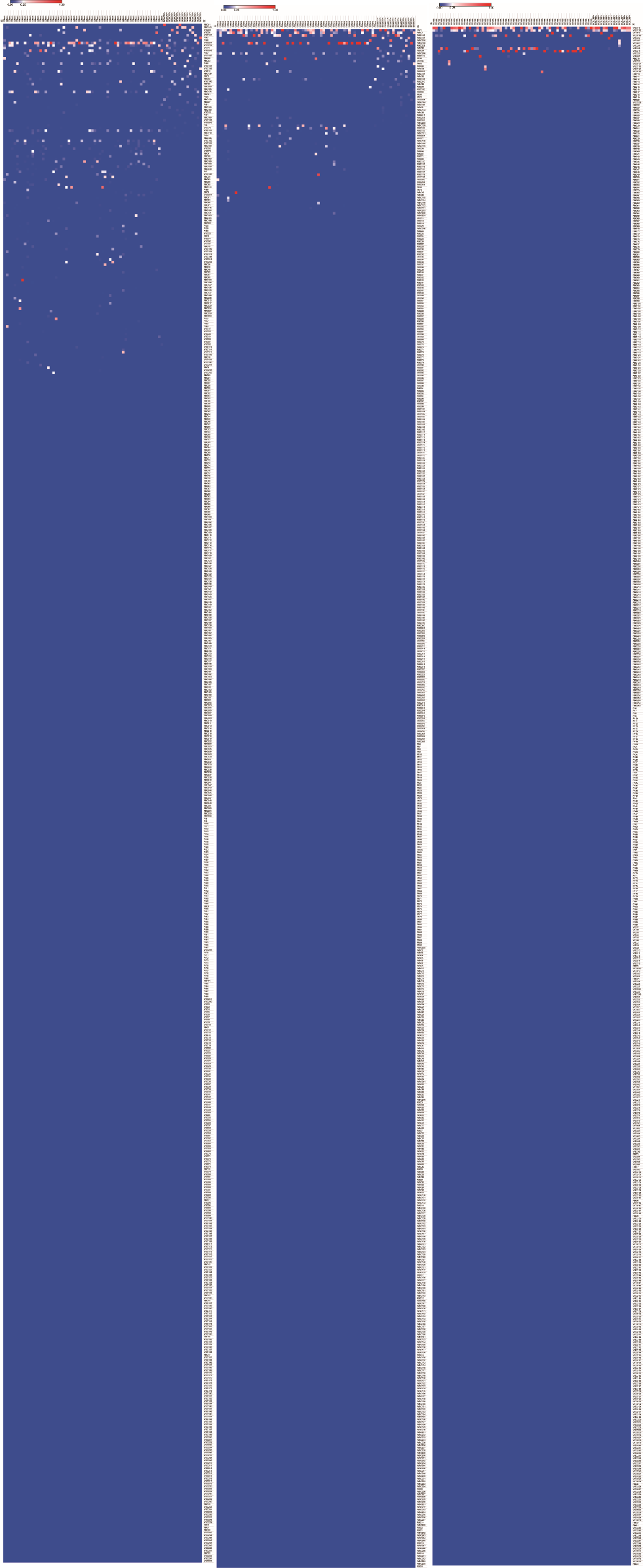


**Supplementary Figure S5.** The all features in the all three heatmaps of the CRP classifier. The color scheme represents heatmap values from 0 to 1. “Predicted outcome others” group, n=62; “Blood culture (BC) positive and CRP ≥10 mg/dL” group, n=15. Left: The first heatmap, which is the least near layer to the CRP output. Middle: The second heatmap, which is the second near layer to the CRP output. Right: The third heatmap, which is the nearest layer to the CRP output.


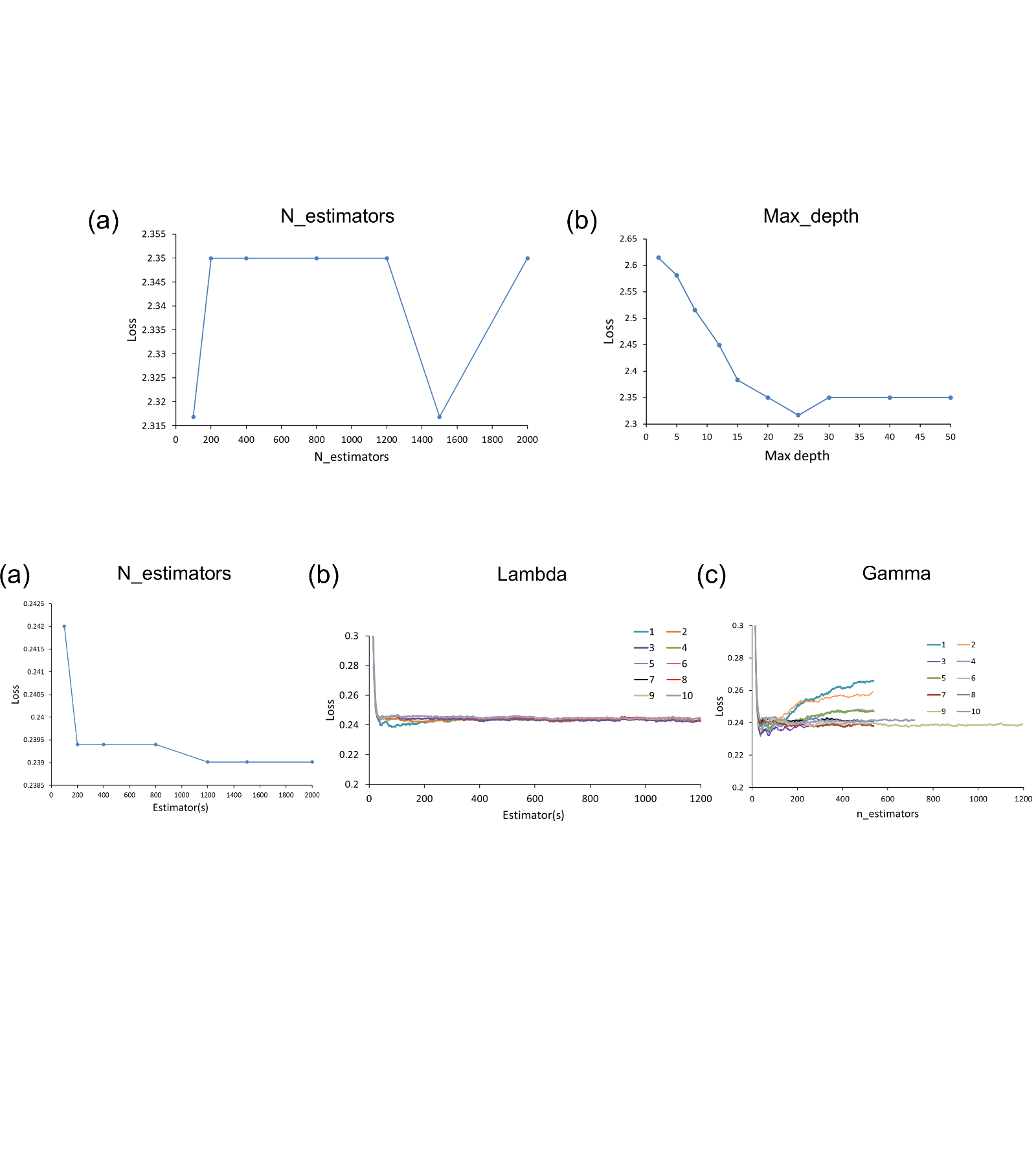


**Supplementary Figure S6.** Learning curve of the hyperparameters in the Random Forest model. (a) Learning curve of the number of the estimators in ensemble learning; we choose n=100 as our estimator. (b) Learning curve of Max_depth hyperparameter; we choose Max_Depth=25 as our training hyperparameter for each classifier.


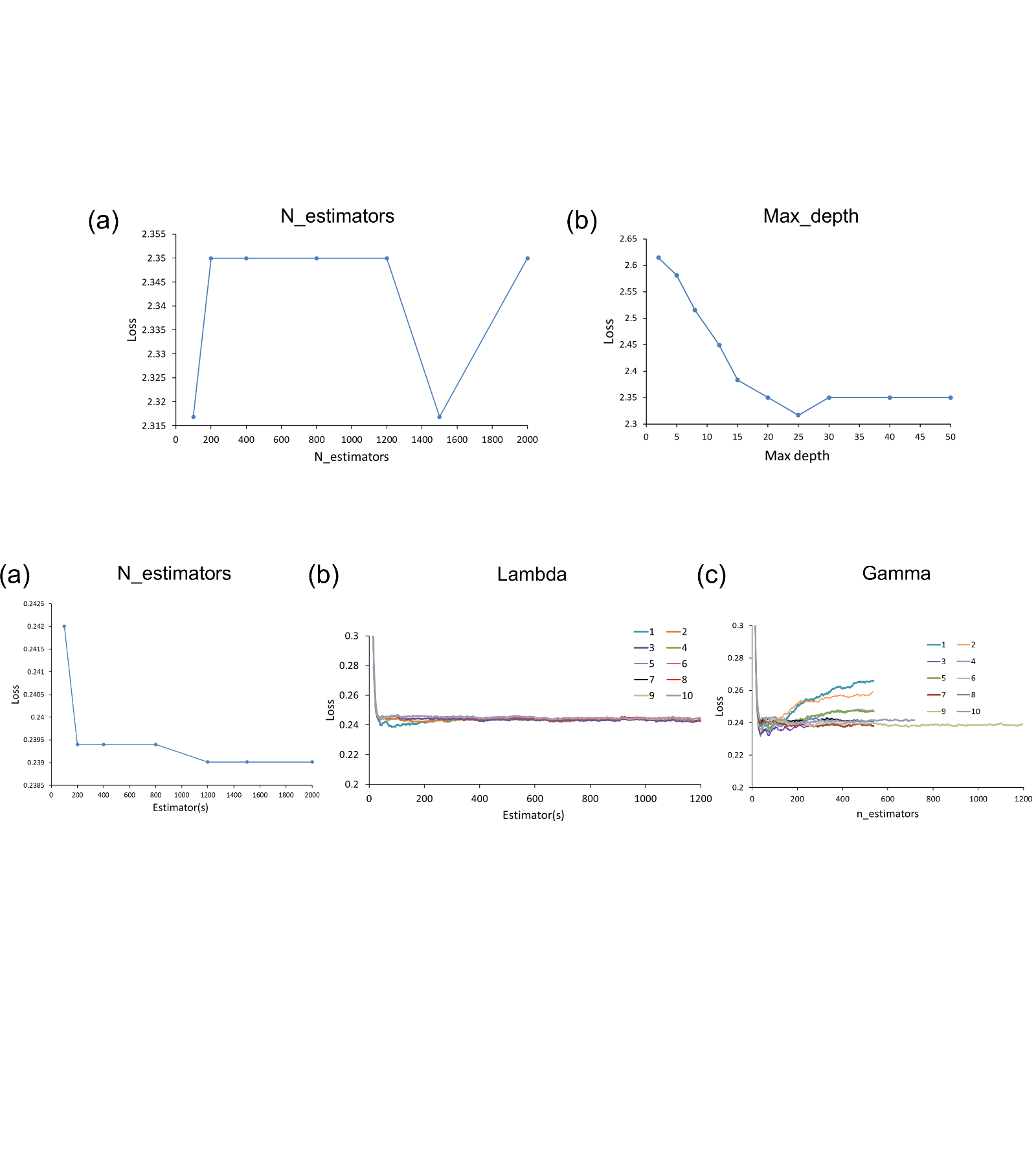


**Supplementary Figure S7.** Learning curve of the hyperparameters in the XGBoost model. (a) Learning curve of the number of the estimators in ensemble learning. Since more estimators may cause overfitting, we choose n=1200 as our estimator. (b) Learning curve of the lambda hyperparameter; we choose lambda=1 as our training hyperparameter for each classifier. (c) Learning curve of the gamma hyperparameter; we choose gamma =1 as our training hyperparameter for each classifier.


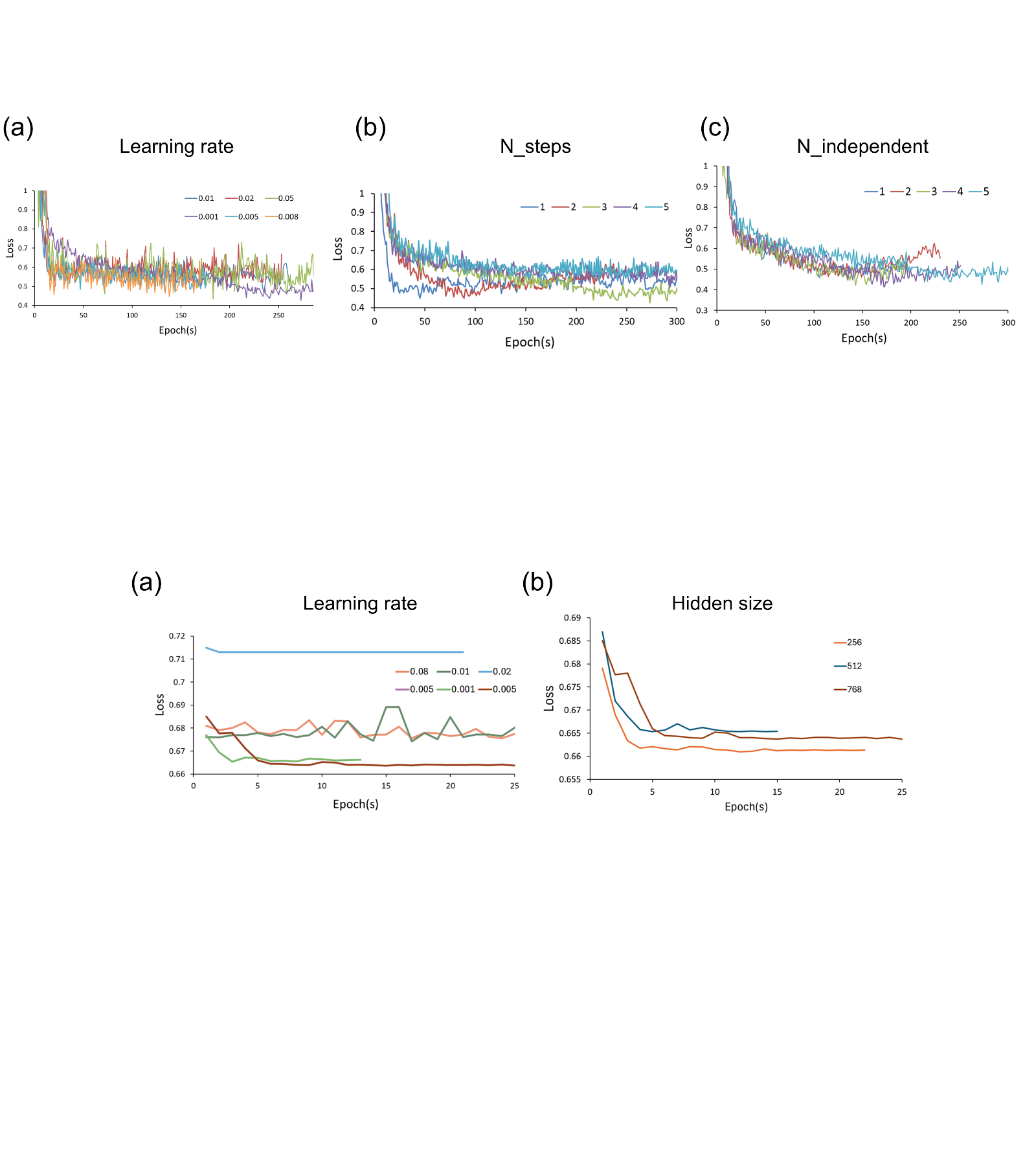


**Supplementary Figure S8.** Learning curve of the hyperparameters in the TabNet model. (a) Learning curve of learning rate; we choose learning rate = 0.001 as our training hyperparameter for each classifier. (b) Learning curve of N_steps; we choose n=3 as our N_step hyperparameter for each classifier. (c) Learning curve of N_independent; we choose n=5 as our N_independent hyperparameter for each classifier.


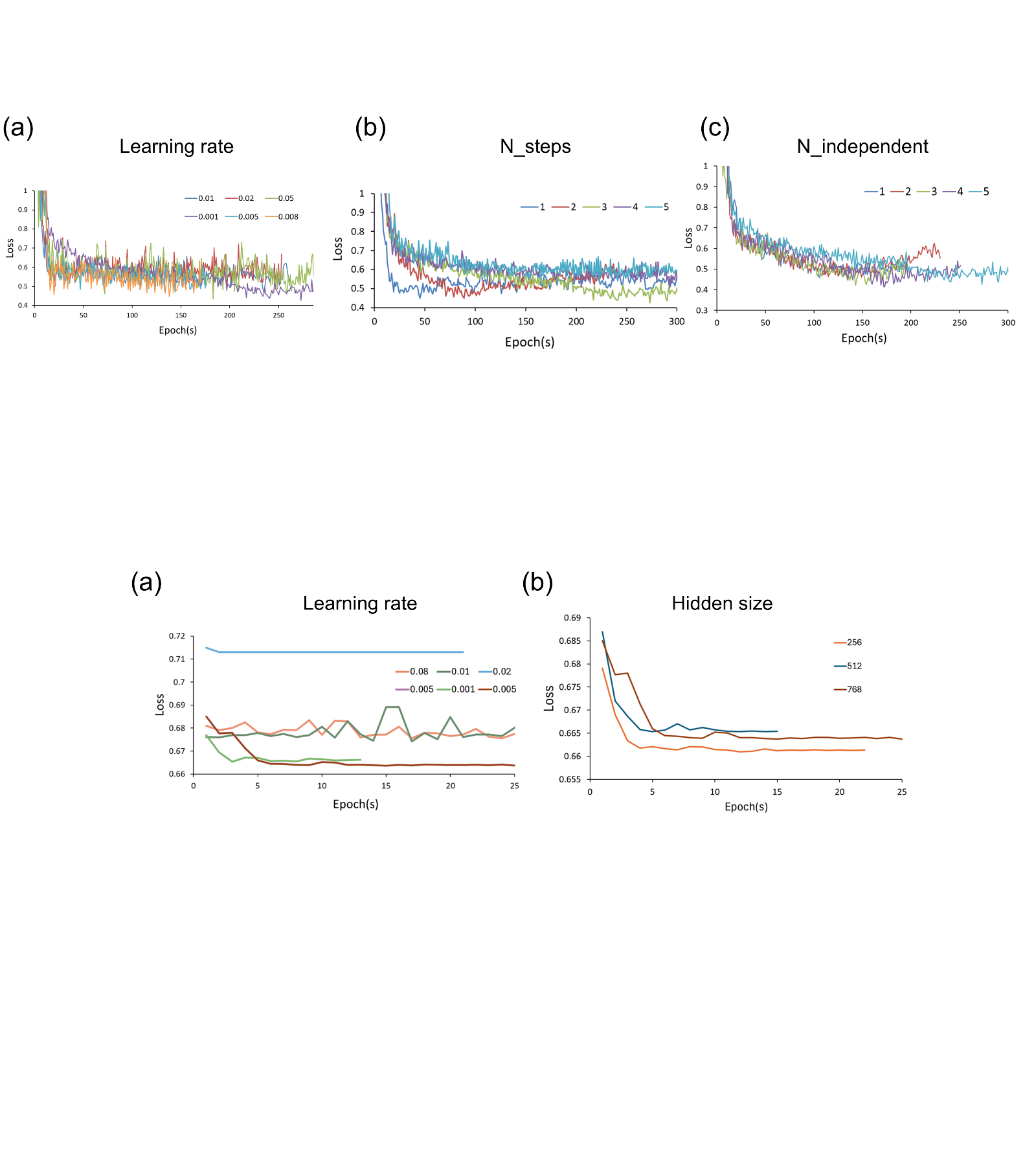


**Supplementary Figure S9.** Learning curve of the hyperparameters in LSTM model. (a) Learning curve of learning rate; we choose learning rate = 0.005 as our training hyperparameter for each classifier. (b) Learning curve of hidden size; we choose Hidden size = 256 as our hyperparameter for each classifier.
